# New Cupping Apparatus

**Published:** 1853-06

**Authors:** 


*New Cupping Apparatus.*—Dr. O. H. Needham, of the city of New York, has contrived a new cupping apparatus, which appears to be greatly superior to that in common use, and promises, to a considerable extent, to take the place of leeches. The improvement consists: *First*, in effecting the exhaustion of the cups by an instrument, attached thereto by means of a flexible tube, which is worked like a bellows. The operation can readily be performed by the patient. The appendage is light and small, requiring very slight effort in pumping out the air. *Second*, the cups are made of glass, to which are attached rims of India rubber. The adhesion to the skin is by means of the latter, being less painful, and at the same time, more complete than with cups made entirely of glass. The India rubber extremities are made with different shapes and sizes, so as to be readily applied to any part, and even accommodated to inequalities of surface. They may thus be adapted to parts where the ordinary cup is inadmissible, taking the place of leeches in such cases.

Connected with the apparatus is a breast-pump, which is found to be more effectual and less painful than the instruments in common use.

We have been much gratified by an examination of these apparatus, and we cannot doubt that they will come into general use.

